# Bioactivity of Root Endophytic Freshwater Hyphomycetes *Anguillospora longissima* (Sacc. & Syd.) Ingold

**DOI:** 10.1155/2014/707368

**Published:** 2014-10-14

**Authors:** S. C. Sati, Lokendra Singh

**Affiliations:** Department of Botany, Kumaun University, DSB Campus, Nainital 263 002, India

## Abstract

*Anguillospora longissima*, isolated from root as endophytic freshwater hyphomycetes, was evaluated for its bioactivity (antibacterial potential) against five bacterial strains, namely, Gram-positive (*Bacillus subtilis* MTCC 121) and Gram-negative (*Agrobacterium tumefaciens* MTCC 609, *Escherichia coli* MTCC 40, *Erwinia chrysanthemum,* and *Xanthomonas pseudomonas*). Antimicrobial activity was assessed by measuring the zone of inhibition with preliminary and secondary antimicrobial assays. The applied fungus was found significant for all tested bacterial strains as showen by their zone of inhibition. In preliminary antimicrobial assay, maximum zone of inhibition was recorded against Gram-negative human pathogenic bacterial strain *Escherichia coli* (23 mm) followed by *Erwinia chrysanthemi* (22 mm), *Agrobacterium tumefaciens* (21 mm), and *Xanthomonas phaseoli* (21 mm), while minimum zone of inhibition was observed against *Bacillus subtilis* (20 mm). In secondary antimicrobial assay, the maximum zone of inhibition was recorded against *Erwinia chrysanthemi* (11 mm) followed by *Agrobacterium tumefaciens* (10 mm), *Xanthomonas phaseoli* (10 mm), and *Bacillus subtilis* (9 mm) and minimum inhibition was found against *Escherichia coli* (8 mm).

## 1. Introduction

A large number of antibiotics have lost their effectiveness against the resistant bacterial strains, mostly by the expression of resistance genes [1]. Fungi are well known to produce antibiotics [2] and continue to be investigated as a source of natural products and secondary metabolites [3] for their potential in medical, industrial, and agricultural use [4–7]. Natural products or byproducts from microorganisms constitute a major source of desirable bioactive molecules. Endophytic fungi appear to be more potent as a good source of metabolites and may produce a plethora of such substances. Natural products from endophytes have been observed to inhibit or kill a wide variety of harmful microorganisms [8]. Exploitation of novel classes of antimicrobial metabolites is increasingly noticeable over recent years and investigation on diversity, ecology, and secondary metabolites of endophytic fungi is continued [9]. Therefore, there might be a good scope of some novel or useful bioactive compounds by screening of endophytic fungi for their antimicrobial activities.

Aquatic hyphomycetes, a group of nonzoosporic aquatic fungi, have not, however, been extensively surveyed as antibiotic producer, perhaps owing to their ecological peculiarities. In contrast to the majority of terrestrial fungi, aquatic hyphomycetes show a variety in characteristic conidial shapes, which range from simple rounded spores to filiform and branched triradiate or tetraradiate spores. Tetraradiate shape is the dominant spores, while filiform shapes are the less common with experimental evidence suggesting that more complex and branched spore shapes resulted in adaptation to turbulent aquatic environments [10]. There are only few reports to describe the antimicrobial potential of root endophytic aquatic hyphomycetous fungi and on the production of their diffusible inhibitory substances [11–13]. The study on isolation and structural determination of antimicrobial compounds from* Anguillospora longissima* and* A. crassa* has resulted in the discovery of some novel metabolites [14]. Quinaphthin, a new antimicrobial compound, has also been described from the aquatic hyphomycetes* Helicoon richonis* (Boud.) Linder [15, 16]. The present investigation was focused on the isolation and antibacterial assessment of root endophytic freshwater fungus* Anguillospora longissima* against some selected pathogenic bacterial strains.

## 2. Material and Methods

### 2.1. Collection and Isolation of Root Endophytic Fungi

Roots of aquatic and riparian plants were collected from the streams and their catchment areas of Nainital, Kumaon Himalayas, Uttarakhand, India. Fresh and healthy roots from the living plants were carefully plucked, kept in presterilized polybags, and brought to laboratory. Samples were thoroughly washed with running tap water (2–4 hrs), then with sterile water (5–7 minutes), and finally with 2% sodium hypochlorite solution (1-2 minutes) to remove adhering free living microbes of the root surfaces. Samples were then cut into small pieces (1-2 cm long) and placed into Petri dishes containing ≈20 mL of sterile water. The root segments containing Petri dishes were incubated at 20 ± 2°C for 3–7 days and observed regularly. Initial observations were made with low power and studied further with high power of a compound microscope to confirm the mycelium growth and liberated conidia in water culture. The floating conidia were picked up by sterile needle and studied further by making permanent slides. Pure cultures were prepared by transferring the conidia onto the 2% malt extract agar (MEA) and maintained in the form of MEA slants at 4°C.

### 2.2. Preliminary Antimicrobial Assay

For antimicrobial assay,* Bacillus subtilis* MTCC 121 was used as Gram-positive and* Agrobacterium tumefaciens* MTCC 609,* Escherichia coli* MTCC 40,* Erwinia chrysanthemi*, and* Xanthomonas phaseoli* were used as the Gram-negative test bacteria. The Nutrient Agar medium was used for cultivation of the test bacteria, and the malt extract agar medium was used for cultivation of* Anguillospora longissima*. The medium for testing bacteria was poured into Petri dishes and inoculated with 200 *μ*L of the bacterial suspension (after incubation for 2 days at 37°C). Then, the inoculum was spread by a sterile glass rod (L-shaped) on the surface of the medium. Mycelium discs (5 mm diameter) of isolated fungus (7-day-old culture, grown on MEA medium plate at 25°C) were cut using a sterile borer and placed onto the surface of the above medium seeded with test organisms [17]. The plates were refrigerated at 4°C overnight for complete diffusion of antibiotics; thereafter, they were incubated at 37°C for 16 h. The diameter of the inhibition zone was measured and the average of three repeated agar discs was taken to assess the strength of antimicrobial activity.

### 2.3. Secondary Antimicrobial Assay

The fungal strain* Anguillospora longissima* was inoculated into 250 mL conical flasks containing 50 mL of ME broth medium and was shaken on a thermostatic shaker with the rotary speed of 180 rpm at 25°C. After 7 days, the culture was centrifuged at 5000 rpm at 4°C to isolate the mycelia and the broths. The mycelia were extracted with 100 mL of methanol and the broth was extracted with 150 mL of ethyl acetate to yield the methanol extract and the ethyl acetate extract, respectively. These two extracts were filtered, combined, and evaporated to dryness. The resulting crude extract was finally dissolved in 2.5 mL of methanol for assay. In a clean air bench, extract was added using a pipette to a sterile paper disc (5 mm diameter, Whatman number 1), which was air dried and placed on the surface of the medium seeded with test organisms [18]. Likewise, the plates were incubated and measured for inhibition zones. The average of three repeated paper discs was taken to evaluate the activity. The broad spectrum antibacterial agent Gentamycin (dose: 30 mcg/disc) and methanol were used as positive and negative control.

## 3. Results and Discussion

### 3.1. Morphological Features of Isolated Root Endophytic Fungus

The isolated fungus is a root endophytic aquatic fungus with branched septate mycelium. Conidiophores are 90–100 × 3-4 *µ*m in size and bear conidia singly at the tips. The conidia are hyaline, filiformis, curved or sigmoid with 5–14 septation and 140–221 × 3–5 *µ*m in size being 4-5 *µ*m wide at middle and tapers toward the ends. So, on the basis of morphological analyses, the isolated fungus was identified as* Anguillospora longissima* (Sacc. & Syd.) Ingold. The culture was maintained on 2% MEA for further work.

The isolated fungus* Anguillospora longissima* was recovered from* Equisetum* sp. as a root endophyte and was applied against plant and animal pathogenic bacterial strains to evaluate its antibacterial potentiality.

### 3.2. Preliminary Antimicrobial Assay

**Figure 1:**
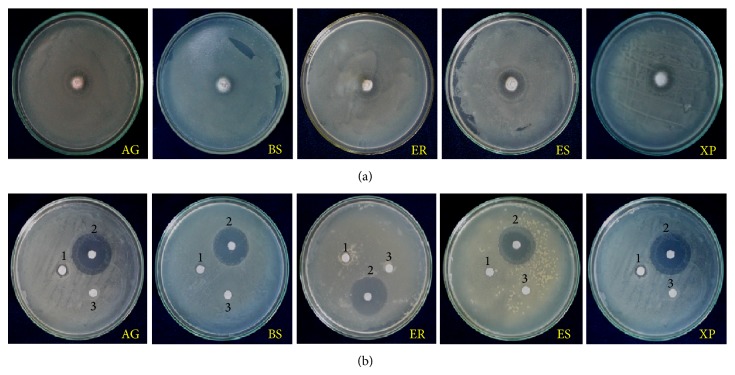
Antibacterial activity of isolated root endophytic fungus* A. longissima* against pathogenic bacteria during preliminary (a) and secondary (b) antimicrobial assays. AG:* Agrobacterium tumefaciens*; BS:* Bacillus subtilis*; ER:* Erwinia chrysanthemi*; ES:* Escherichia coli*; XP:* Xanthomonas phaseoli*; 1: fungal extract; 2: positive control (Gentamycin); 3: negative control (methanol).

**Figure 2:**
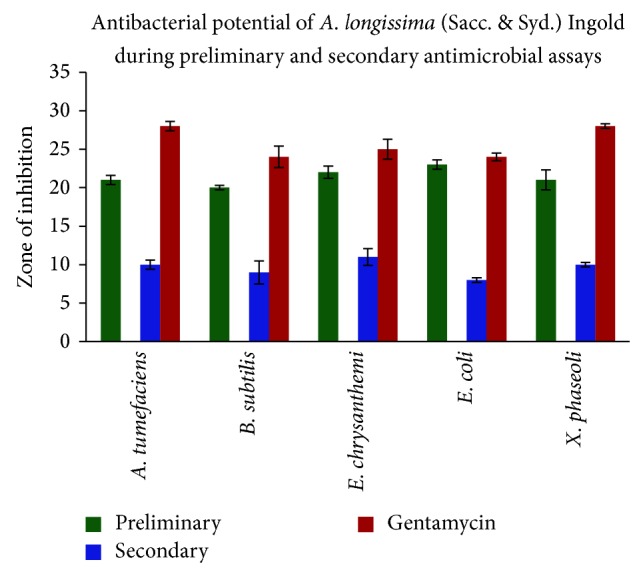
Antimicrobial activity of* Anguillospora longissima* in preliminary and secondary antimicrobial assays (disc diffusion method). Positive control: Gentamycin (dose: 30 mcg/disc) for antibacterial test and negative or blank control: methanol.

Discs (5 mm) of fungal mycelium were used for preliminary antimicrobial assay. The isolated root endophyte aquatic fungus* Anguillospora longissima* possesses strong inhibitory activities against all test organisms (Figure 1). Maximum zone of inhibition was observed against Gram-negative animal pathogenic bacterium* Escherichia coli* (23 mm) followed by* Erwinia chrysanthemi* (22 mm),* Agrobacterium tumefaciens*, and* Xanthomonas phaseoli* (21 mm), while minimum inhibition zone was observed against Gram-positive bacterium* Bacillus subtilis* (20 mm). The applied fungus has shown inhibitory activity against both Gram-positive and Gram-negative bacterial strains (Figure 2; Table 1).

### 3.3. Secondary Antimicrobial Assay

Seven-day-old culture of* Anguillospora longissima*, grown in MEB (malt extract broth), was harvested and the ethyl acetate-methanol extracts were rescreened for antimicrobial activity using paper disc agar diffusion method. The results were summarized in Table 1. As evident from the table, the isolated root endophytic fungus* A. longissima* is indeed able to produce antimicrobial substances responsible for the growth inhibition of all the tested bacterial strains (Table 1; Figure 2). In secondary antimicrobial assay, the maximum zone of inhibition was recorded against the bacterial strains* Erwinia chrysanthemi* (11 mm) followed by* Agrobacterium tumefaciens* and* Xanthomonas phaseoli* (10 mm for both), while minimum zone of inhibition was recorded against human pathogenic bacterium* Escherichia coli* (8 mm).

The aquatic fungus* Anguillospora longissima* was isolated as root endophyte from a riparian pteridophytic plant* Equisetum* sp., from Nainital, Kumaon Himalaya, India. The bioassay data has shown that root endophytic fungus was able to inhibit all the tested bacterial strains (Table 1); however, the potentials varied from species to species. The fungus is well known to produce metabolites and for a novel compound “Anguillosporal” [14]. It has been attributed that the existence of endophytes is probably originated from nutritional competition between endophytes and other organisms [19] and this phenomenon was speculated to be derived from some chemicals or antibiotic substances by these fungi for their defense and adjustment with hosts in freshwater streams ecosystem. In the present investigation, the studied root endophytic fungus* A. longissima* was also isolated from freshwater habitat that showed a definite antibacterial property against the tested pathogenic bacteria. The antimicrobial potential of endophyte might be attributed to the secretion of secondary metabolites, that is, antibiotics or enzymes which were responsible for inhibition in bacterial growth.

The antimicrobial potential of freshwater hyphomycetes has also been studied earlier by a few workers. Chamier et al. [20] reported inhibition of bacteria by some aquatic hyphomycetes in their field experiments. Shearer and Zare-Maivan [12] reported eight species of aquatic fungi (three of them are aquatic hyphomycetes) were able to inhibit the growth of other fungi, suggesting that they produce certainly some antibiotic substances. Gulis and Stephanovich [21] found the growth inhibition of some bacterial and pathogenic fungal strains by using freshwater hyphomycetes, including* A. longissima*. Recently, Sati and Arya [22] and Arya and Sati [13] also reported antimicrobial potential of five freshwater hyphomycetes against seven pathogenic fungi and five bacteria. Xiao et al. [23] isolated antimicrobial compounds from the fungus* Pestalotiopsis photiniae* and evaluated inhibitory potential of this fungus against some plant pathogenic fungi. Similarly,* Massarina aquatica* was found inhibiting growth of the yeasts* Candida albicans* [24] and* Sporobolomyces roseus* [25].

## 4. Conclusion

In the present study, the applied fungus* A. longissima* which was isolated as root endophyte might produce some antibiotic substances as found in preliminary antibacterial assay and confirmed by secondary antimicrobial assay. These antibiotic substances were responsible for inhibiting the growth bacterial strains. The living mycelium disc (5 mm) and crude extract both were found effective for the inhibition of microbes as shown in the present study, a considerable inhibitory property against all five tested bacterial strains. On the basis of these findings, it could also be concluded that the endophytic freshwater aquatic hyphomycetous fungi might be an important source of active pharmacologic compounds. However, the commercial implication for the production of desirable antimicrobial compounds by the endophytic fungi still remains a future goal [26]. Nevertheless, the taxonomy and bioactivities of root endophytic freshwater aquatic hyphomycetes might be of some value in promoting our understanding of aquatic hyphomycetes in freshwater ecosystem.

## Figures and Tables

**Table 1:** Antibacterial activity of root endophytic aquatic fungus *Anguillospora longissima* against pathogenic bacteria during preliminary and secondary antibiotics assays.

Pathogenic bacteria	Diameter of inhibition zone (mm)∗		
	Preliminary	Secondary	Gentamycin
*Agrobacterium tumefaciens *	21 (±0.6)	10 (±0.6)	28 (±0.6)
*Bacillus subtilis *	20 (±0.3)	9 (±1.5)	24 (±1.4)
*Erwinia chrysanthemi *	22 (±0.8)	11 (±1.1)	25 (±1.3)
*Escherichia coli *	23 (±0.6)	8 (±0.3)	24 (±0.5)
*Xanthomonas phaseoli *	21 (±1.3)	10 (±0.3)	28 (±0.3)

^*^Mean of three replicates and ±standard error mean (SEM).
